# Raman, TEM, EELS, and Magnetic Studies of a Magnetically Reduced Graphene Oxide Nanohybrid following Exposure to *Daphnia magna* Biomarkers

**DOI:** 10.3390/nano12111805

**Published:** 2022-05-25

**Authors:** Juan A. Ramos-Guivar, Jacquelyne Y. Zarria-Romero, Yamerson Canchanya-Huaman, Jorge Andres Guerra, Noemi-Raquel Checca-Huaman, Isabel-Liz Castro-Merino, Edson C. Passamani

**Affiliations:** 1 Grupo de Investigación de Nanotecnología Aplicada para Biorremediación Ambiental, Energía, Biomedicina y Agricultura (NANOTECH), Facultad de Ciencias Físicas, Universidad Nacional Mayor de San Marcos, Av. Venezuela Cdra 34 S/N, Ciudad Universitaria, Lima 15081, Peru; jacquelyne.zarria@unmsm.edu.pe (J.Y.Z.-R.); yamerson2016@gmail.com (Y.C.-H.); 2 Departamento de Ciencias, Sección Física, Pontificia Universidad Católica del Perú, Av. Universitaria 1801, Lima 15088, Peru; guerra.jorgea@pucp.edu.pe; 3 Centro Brasileiro de Pesquisas Físicas (CBPF), R. Xavier Sigaud, 150, Urca, Rio de Janeiro 22290-180, Brazil; noemiraquelchecca@gmail.com (N.-R.C.-H.); isabel5cas@hotmail.com (I.-L.C.-M.); 4 Physics Department, Federal University of Espírito Santo, Vitória 29075-910, Brazil; edson.caetano@ufes.br

**Keywords:** nanohybrid recoverage, *Daphnia magna* biomarkers, lethal dose, post-exposure characterization

## Abstract

A ternary nanocomposite made of nanomaghemite, nanoanatase, and graphene oxide has been successfully synthesized using an inorganic coprecipitation approach, and it has been systematically investigated by X-ray diffraction, transmission electron microscopy, and different spectrocopic techniques (electron energy loss, µ-Raman, and ^57^Fe Mössbauer) after interaction with an effluent containing *Daphnia magna* individuals. Specifically, the influence of the nanocomposite over the *Daphnia magna* carapace, administered in two doses (0.5 mg mL^−1^ and 1 mg mL^−1^), has been characterized using µ-Raman spectroscopy before and after laser burning protocols, producing information about the physicochemical interaction with the biomarker. The thermal stability of the nanocomposite was found to be equal to 500 °C, where the nanoanatase and the nanomaghemite phases have respectively conserved their structural identities. The magnetic properties of the nanomaghemite have also been kept unchanged even after the high-temperature experiments and exposure to *Daphnia magna*. In particular, the size, texture, and structural and morphological properties of the ternary nanocomposite have not shown any significant physicochemical modifications after magnetic decantation recuperation. A significant result is that the graphene oxide reduction was kept even after the ecotoxicological assays. These sets of observations are based on the fact that while the UV-Vis spectrum has confirmed the graphene oxide reduction with a localized peak at 260 nm, the 300-K and 15-K ^57^Fe Mössbauer spectra have only revealed the presence of stoichiometric maghemite, i.e., the two well-defined static magnetic sextets often found in the bulk ferrimagnetic counterpart phase. The Mössbauer results have also agreed with the trivalent-like valence state of Fe ions, as also suggested by electron energy loss spectroscopy data. Thus, the ternary nanocomposite does not substantially affect the *Daphnia magna*, and it can be easily recovered using an ordinary magnetic decantation protocol due to the ferrimagnetic-like character of the nanomaghemite phase. Consequently, it shows remarkable physicochemical properties for further reuse, such as cleaning by polluted effluents, at least where *Daphnia magna* species are present.

## 1. Introduction

*Daphnia magna* (*D. magna*) species are environmental biomarker models widely used to study and comprehend the ecotoxicology effects of different inorganic and organic materials (e.g., nanoparticles and pharmaceuticals) on different biological levels, such as the cellular, reproductive, and molecular levels [1,2], the last one often applied to understand genome behavior. From the evolutionary point of view, changes, adaptations, and phenotypic variations are fundamental features to be modeled and studied during an ecotoxicology analysis due to its important location in the trophic chain when nanomaterials are used for water cleaning processes of real effluents [3,4,5]. For instance, recent advances and developments in several nanohybrids for the removal of toxic metals and other organic hazardous materials suggest their final spreading into water bodies, soils, various effluents, and the construction industry [6,7,8,9,10], a condition that requires tests of their ecotoxicological effects. For example, cytotoxic and morphological analyses have been studied [11,12,13,14,15], but lethal concentrations (*LC*_50_, mg L^−1^) need to be established and will depend on the developed nanoadsorbents (nanocomposite).

Regarding this issue, in-situ treatments of lakes and rivers, using magnetic nanohybrids without previous ecotoxicological evaluation, can drastically damage the biota and aquatic population dynamics (i.e., mortality rate, lifetime, and morphological changes as a product of cytotoxicity). As an example, Gökçe et al. [11] have reported the ecotoxicological effects of titanium dioxide (TiO_2_) and zinc oxide (ZnO) nanoparticles (NPs) against *D. magna*. After 96 h of exposition, they have found *LC*_50_ values of 1.8 mg mL^−1^ for TiO_2_ NPs and 0.7 mg L^−1^ for ZnO NPs, yielding a reduction in the reproductive cycle and uptake of the NPs by the digestive system and carapace. On the other hand, Magro et al. [12] have studied the surface reactivity of iron-oxide NPs on *D. magna* adults and embryos, and the main results have shown a prevalence for NPs to deposit onto the carapace biomarker. Moreover, the recuperation of these nanohybrids is another important point to be considered after application in treatments of the environment. Actually, there is a lack of available information about recuperation protocols and of further studies on structural, colloidal and thermal stabilities and magnetic properties of the used nanocomposite. Therefore, the evaluation of these points using different analytic techniques certainly will control the recyclability properties of the nanohybrids and will bring information about their impacts on the environment.

In this work, we have first characterized the structural and magnetic properties of a ternary nanocomposite made of reduced graphene oxide (r-GO) + anatase (TiO_2_) + maghemite (γ-Fe_2_O_3_) and immobilized onto *D. magna* in two different concentrations (0.5 and 1 mg L^−1^), being the first dose (corresponding to the *LC*_50_ value) previously obtained from ecotoxicological experiments [15]. Specifically, we are now bringing new information on the effect of the ternary nanocomposite on the *D. magna*’s DNA, which has been evaluated using an electrophoresis protocol. Regarding the physical properties of the nanocomposite, we have to mention that a systematic investigation has been completed, where we have determined that the excitation laser power of 0.83 mW (used in Raman experiments) has favored the α→γ-Fe_2_O_3_ structural transformation of the magnetic phase of the nanocomposite that already has r-GO deposited onto *D. magna*. In addition, the recuperation process of the nanocomposite (by flocculation and magnetic decantation, and then dried for measurements) has also been investigated, as their physical properties. Specifically, the corroboration of r-GO was studied by the intensity ratio, i.e., it was based on the Raman crystal size estimation and UV-Vis spectrometry. On the other hand, Rietveld refinement, transmission electron microscopy (TEM) images, scanning transmission electron microscopy (STEM), high-resolution transmission electron microscopy (HRTEM), energy-dispersive X-ray spectroscopy (EDS), and electron energy loss spectroscopy (EELS) have allowed a detailed differentiation between the metal oxide phases, and consequently information about their size, texture, and morphological properties as presented in the ternary nanocomposite. The thermal properties have been also studied by thermogravimetric analysis (TG), where the weight loss has suggested the functionalization of the r-GO with the anatase and γ-Fe_2_O_3_ NPs. We finally studied the magnetic properties by zero-field ^57^Fe Mössbauer spectroscopy and VSM measurements that showed fingerprint signals of pure γ-Fe_2_O_3_.

## 2. Materials and Methods

### 2.1. Characterization

The X-ray diffraction (XRD) experiment was performed with an Empyrean diffractometer (Malvern Panalytical, Malvern, UK) at the Brazilian Center for Physics Research, operating with CuKα radiation at wavelength λ = 1.54056 Å emitted by a Cu anode in 45 kV and 40 mA. XRD data were collected in the 2*θ* = 10–80° range using Bragg–Brentano geometry in spinner mode with 0.026° and 2000 s per step. Crystallographic phase identification was performed with Match version 3 software (V3, Crystal Impact, Bonn, Germany) [16]. For the γ-Fe_2_O_3_ phase, the initial parameters were a cubic crystalline structure, space group Fd$\bar{3}$m, and cell parameter a = 8.33 Å (Match entry 900-6317), whilst for the TiO_2_ anatase (Match entry 500-0224), they were a tetragonal crystalline structure, space group I41/amd, a = 3.789 Å, and c = 9.537 Å. The software FullProf Suite (Gif sur Yvette Cedex, France, version January 2021) was employed for the Rietveld refinement, where the Thompson–Cox–Hastings (TCH) pseudo-Voigt axial divergence asymmetry function was used as a function of the diffraction peak profile. Finally, the instrumental resolution function (IRF) of the diffractometer has been obtained from the aluminum oxide (Al_2_O_3_) standard with Caglioti parameters, U = 0.0093, V = −0.0051, and W = 0.0013 [17].

The µ-Raman spectra were carried out at ambient conditions in a Renishaw inVia Raman microscope (Edinburgh, UK) in reflection geometry under 785 nm excitation wavelength with an initial laser power of 82.8 mW over the sample. The employed optical objective was of 50× magnification, with a laser spot of 1.3 µm. The protocol to increase the laser power had two steps: (i) measurements before burning were performed with a laser power of 1% and 5% of the initial value during intervals of 60 and 240 s, and (ii) after-burning µ-Raman measurements were performed, such as: the laser power percentage of 10% was first kept for 60 s of exposure, then the µ-Raman spectra were collected following step (i). The following nomenclature was used for the studied samples: DM1 for the *D. magna* immobilized onto glass substrate at 0 mg mL^−1^ dose, DM2 for the *D. magna* over the glass substrate treated with a 0.50 mg mL^−1^ dose of the ternary nanocomposite, and DM3 for the *D. magna* over the glass substrate treated with a 1 mg mL^−1^ dose of the ternary nanocomposite. Immobilization of the *D. magna* over the glass substrate was achieved by first rinsing the species in 90% ethanol and then placing them on a glass slide. Subsequently, they were dried in an oven at 50 °C for 24 h. Thermogravimetry (TG) measurements were carried out using Shimadzu equipment (Kyoto, Japan), where the samples were heated from RT to 500 °C in a synthetic air atmosphere (flux rate of 50 mL min^−1^) with a heating rate of 10 °C/min. Absorbance UV Vis spectrum was taken at room temperature (RT) using an AVANTES spectrometer (Apeldoorn, the Netherlands) in the region from 200–300 nm, an integration time of 30 ms and average of 100 repetitions were used to collect the spectrum, then the data were exported to be processed with the AvaSoft8 software version.

The ^57^Fe Mössbauer spectrum at 15 K and RT (300 K) were collected in transmission mode by employing a conventional spectrometer working in sinusoidal velocity sweep and with a 40 mCi source of ^57^Co in Rh matrix. For the low-temperature measurement, the source was kept at RT, and the absorber temperature was decreased to 15 K using a Janis closed-cycle setup. The powder absorber was placed into nylon sample holders, and its effective thickness was selected to be equivalent to ca. 0.1 mg ^57^Fe per cm^2^.

Zero-field-cooling (ZFC) and field-cooling (FC) magnetic hysteresis loops (*M*(*H*) loops) were recorded by a vibrating sample magnetometer operating in a dynacool setup for a maximum field of 7 T. The FC experiment was performed with a cooling field of 1 T and a sweep field of ±7 T (the FC experiments were performed to check for possible exchange bias effects).

### 2.2. TEM Characterization

Average size, distribution, and morphology were investigated by electron microscopy (EM) with three modes: transmission (TEM), scanning (STEM), and high-resolution (HRTEM) using a JEOL 2100F (Tokyo, Japan) instrument operated at an accelerating voltage of 200 kV and equipped with a CMOS camera to acquire TEM and HRTEM images. Lattice fringes were measured from the fast Fourier transform of HRTEM images, using a Gatan Digital Micrograph (GATAN Inc., Pleasanton, CA, USA). STEM images were obtained using a high angular annular dark field (HAADF) field detector. The microscope is equipped with accessories for energy-dispersive X-ray spectroscopy (EDS) and electron energy loss spectroscopy (EELS) (EELS-GIF Tridiem GATAN). The elemental compositions were investigated by EDS and EELS to evaluate the atomic composition at the nanoscale using the mode STEM. EELS experiments were conducted in the STEM imaging mode using a spot size of 0.7 nm. The spectrometer aperture was 5 mm. The energy resolution measured by the FWHM of the zero-loss peaks was approximately 1.8 eV.

### 2.3. Culture of D. magna

The cladocere was cultivated in optimal normal conditions where it would not generate damage or alterations, an important parameter to be controlled since it has been reported that the cultivation process can also cause stress to individuals [18,19,20]. Specifically, the present process occurred considering several steps, such as: first, the circadian rhythm was taken into consideration, and then the individuals were submitted to eight hours of light and sixteen hours of darkness, fed a mixture of microalgae throughout. The fish tanks, used for the culture stocks, were of different volumes depending on the number of individuals that developed. The exoskeleton molts were daily removed, and the individuals were transferred to new tanks every three days.

### 2.4. Effects of Ternary Nanocomposite in D. magna’s DNA

A concentration of nanoparticles of 0.55 mg mL^−1^ was added at which *D. magna* die at 50% (*LC*_50_) [15] to identify how much it could be damaging the DNA of individuals, while in another tank, the *D. magna* were kept in their optimal conditions, as a negative control.

### 2.5. DNA Extraction, Purification, and Quantification

The extraction of nucleic acids from living organisms is a routine process. However, when working with chitinous animals, we must be very careful since the remains of chitin are in the final extracted sample alter the results, hence generating a false increase in DNA concentration because the chitin has an absorbency index of 260 nm, like DNA does [21]. Consequently, when we carry out subsequent experiments, the actual DNA may not be enough for the analysis. Indeed, DNA extraction protocols may vary depending on the kit or how it has been standardized. For example, while some authors have exposed and left it overnight with proteinase K, temperature shocks, and liquid nitrogen, other authors have used sonication to liberate cellular tissues from the chitin shell [21,22].

After a successful DNA extraction process, the DNA can be used for various molecular tests, gene amplification, or others. However, the integrity of the double-strand, its size, and its quantity are important parameters to be considered in order to have successful molecular results. Thus, these types of protocols are already well standardized [23], as are the interpretation of their results [23]. The best part of this technique is that it allows the DNA fragments to be separated according to their size or degree of fragmentation, generating a homogeneous migration capable of being measured in a way comparable to a ladder. Therefore, we currently have a wide variety of markers or fluorophores that are interspersed with it, i.e., DNA to evidence its presence under certain types of wavelength light [24]. *D. magna* were maintained in a 8:16 h light:dark photoperiod and a temperature of (20 ± 1) °C. Specifically, ten juvenile individuals were taken from the negative control and another ten individuals from the submitted treatment, and were homogenized separately in glass tubes, both with 0.5 mL of lysis buffer (obtained from the Thermo Scientific GeneJET Genomic DNA Purification Kit from Waltham, MA, USA) that was sonicated two times at 40 GHz. After DNA extraction, the DNA was cleaned or purified with the Zymo Research DNA Clean & Concentrator Kit to later be able to concentrate and quantify it in order to verify its concentration for Qubit^®^ and the fluorometric technique, as well as its integrity. Isolation and purification with this kit ensure the high quality and integrity of our sample. Therefore, the kit ensures that it provides the ideal performance for different applications, including electrophoresis. On the other hand, the DNA is obviously not completely obtained in its original size, due to the reagents to which it has been subjected; however, if in addition to this, the DNA of the sample presents breaks or fragmentations in the double chain of the DNA, so it will be obtained in smaller sizes [24].

### 2.6. Integrity of DNA for Electrophoresis

**Figure 1:**
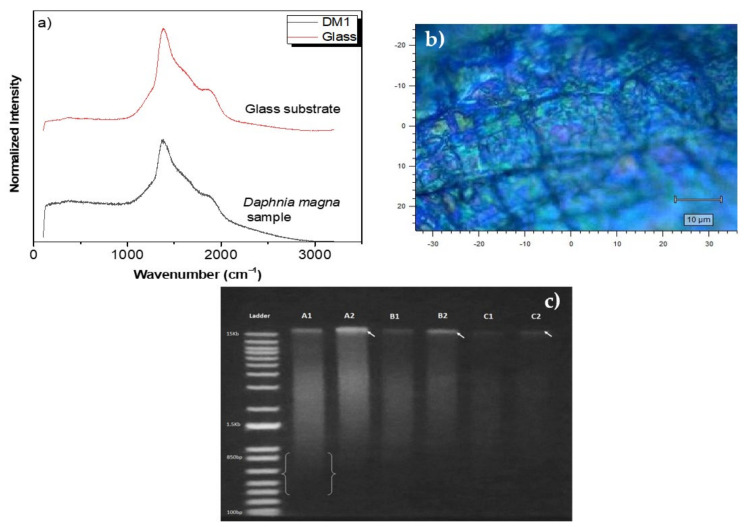
(**a**) µ-Raman spectra for glass substrate and *D. magna* immobilized on the glass substrate (DM1 sample, 0 mg mL^−1^), showing the typical glass-substrate-related luminescence, (**b**) micrograph image of the *D. magna* carapace, and (**c**) gel electrophoresis for different concentrations of DNA from *D. magna*.

The electrophoresis technique is based on generating a mesh, which can vary in density according to the amount of agarose used. Through this mesh, the DNA fragments will be transferred in the direction of the negative pole to the positive pole of the equipment. Since DNA has a negative charge, this technique takes advantage of the properties of the studied molecule [24]. For the electrophoretic run, the agarose gel was made at 1% concentration (0.5 g in 50 mL of 1% TAE buffer) and under standard laboratory conditions (150 V for 30 min). These conditions allow the mobility of small, medium, and large DNA fragments obtained in the previous isolation for the two groups evaluated: the group exposed to the nanocomposite and the control. The sample with smaller DNA fragments runs first, and the sizes can be observed and compared to that of the first position of the gel, which is seen on the ladder (first left column) with the molecular weights (see Figure 1c). In this way, in the upper part of the gel, we have the largest fragments trapped in the mesh, whereas the smallest ones that could pass through it easily are in the bottom.

## 3. Results and Discussion

### 3.1. µ-Raman Analysis and DNA Quantification for Electrophoresis Tests

Figure 1a shows the µ-Raman spectra related to the glass substrate (upper spectrum) exhibiting its typical luminescence under 785 nm excitation and *D. magna* immobilized on the glass substrate (lower spectrum). At a first glance at both graphs, they show a great similarity (not apparent differences), i.e., only two pronounced bands (located at 1384 cm^−1^ and 1871 cm^−1^) are observed; consequently, these are mainly attributed to the glass substrate. In other words, subtracting these spectra, no significant peaks related to the main Raman modes of the *D. magna* species [25] are observed. On the other hand, looking at the micrograph where *D.magna* is present (Figure 1b), it displays the typical carapace for this biomarker [25], confirming its presence on the substrate (its relative amount may be small to give a distinguishable µ-Raman spectrum).

To corroborate the integrity of the specie, we performed electrophoresis tests before and after we determined the *LC*_50_ value. As previously studied, the 24 h of nanocomposite exposure causes death of 50% the biomarker population [15]; thereby, the survivors were separated in clean water for 2 h, and then the sample proceeded to DNA extraction. After the successful extraction of the DNA (applying the DNA cleaning and concentration kit), we obtained a DNA of 60 ng mL^−1^ for both, the treatment and the negative control samples, as displayed in Table 1. The electrophoresis photograph was obtained where the amount of fragmented or smaller DNA evidenced in the second lane is evident.

From Figure 1c, it can be observed that the A1 sample presents fragments below 850 bp (between the brackets), whereas the A2 sample displays a thick line at the beginning indicated by the arrow, showing the size of the DNA fragments obtained. Thus, it can be inferred that they are trapped in the initial hole and do not present very short fragments. Similarly, it occurs between B1 and B2 and C1 and C2, where the arrows indicate the areas where we found the largest DNA fragments. The column, where smearing is visualized, is only clear in the A1 sample due to the concentration of DNA used, and the others have lower concentrations for better visualization and resolution. Additionally, the ternary nanocomposite fortunately does not alter the DNA quantification as compared to the negative control, suggesting that, at this molecular level, the *D. magna*’s integrity is kept.

**Figure 2:**
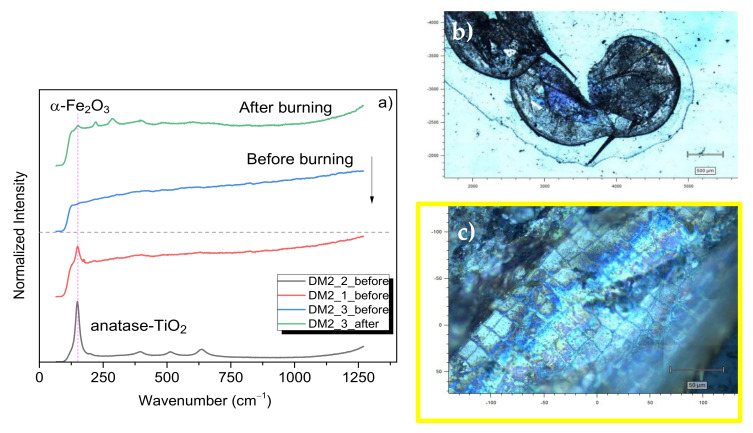
(**a**) Before- and after-burning µ-Raman spectra for the DM2 sample, 5× and (**c**) 50× are two distinct magnifications representing the DM2 sample before burning. The yellow box in (**c**) is a zoomed region of (**b**), where the 1, 2, and 3 index represents the exact illuminated position for the collected spectra given in (**a**).

On the other hand, ecotoxicological experiments were also performed at concentrations of 0.5 mg mL^−1^ and 1 mg mL^−1^, with the aim of characterizing the ternary nanocomposite on the *D. magna* carapace, and hence of corroborating the ternary nanocomposite’s integrity after the studied exposure concentrations. The µ-Raman spectra were collected before and after burning the sample by increasing the laser power, as described in the experimental section. In the case of 0.5 mg mL^−1^ (DM2) (Figure 2a), the main Raman mode at 147 cm^−1^ is seen for the anatase phase in spots 1 and 2 (Figure 2b,c) selected for measurements, while in spot 3, there was not enough signal to collect the µ-Raman spectrum. Specifically, in spot 1 (Figure 2), it is also observed that the main Raman modes for anatase are located at 195 cm^−1^, 393 cm^−1^, 512 cm^−1^, and 637 cm^−1^, which are assigned to the E_g_, E_g_, B_1g_, A_1g_, and E_g_ vibration Raman modes of pure TiO_2_ nanocrystals [26,27]. These results mean that the anatase’s structure is kept after the ecotoxicological experiments for 24 h of exposure, i.e., it shows chemical stability in the tested environment. In addition, as it is notorious, no optical Raman modes of rutile and/or γ-Fe_2_O_3_ phases were observed for the presented conditions. On the other hand, after the burning conditions, the µ-Raman spectra revealed the presence of an α-Fe_2_O_3_ phase, specifically the two main Raman modes at 220 cm^−1^ and 287 cm^−1^ associated with the A_1g_ and E_g_ groups [28]. Again, this observation is also an indication of the presence of a γ-Fe_2_O_3_ phase before burning and not seen previously due to the low Raman signal associated with the γ-Fe_2_O_3_ phase conditions. This after-burning measurement corresponds to a laser power of 0.83 mW, which is above the threshold for the adequate detection of γ-Fe_2_O_3_ (0.1 mW, λ = 785 nm) and the local thermal formation of the α-Fe_2_O_3_ phase, as previously discussed in the literature [29]. In addition, the anatase phase remained with its main mode located at 147 cm^−1^. It is important to highlight that no measurements were performed above 1200 cm^−1^ in order to avoid a misunderstanding of the r-GO overlapped by the glass broad peaks.

**Figure 3:**
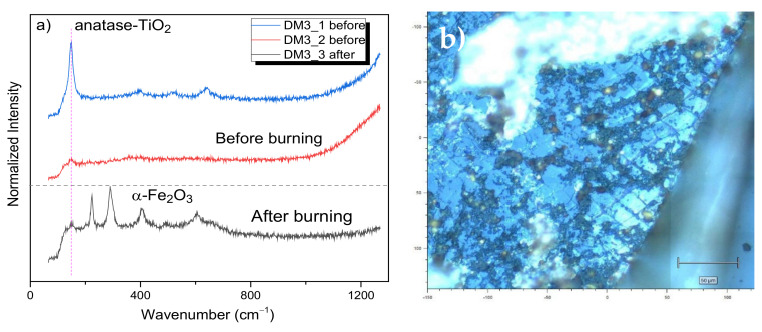
(**a**) Before- and after-burning µ-Raman spectra for the DM3 sample, (**b**) micrograph representing the DM3 sample before burning. 1 is a measurement in a bright point, whilst 2 is a measurement in a dark/brownish place.

For a concentration of 1 mg mL^−1^, the combined ternary nanocomposite exhibited the main Raman modes for anatase before and after burning, as is depicted in Figure 3a. Nevertheless, the after-burning spectrum for 1 mg mL^−1^ shows a better crystallized α-Fe_2_O_3_ phase as compared to the results shown for the *LC*_50_ concentration. In general, we have not detected other Raman modes different from anatase or γ-Fe_2_O_3_ nanocrystals or even the α-Fe_2_O_3_ phase (which is caused by the laser exposition and is not due to the biological assays). In principle, the results corroborate the fact that the metal oxide nanophases do not interact chemically with the *D. magna* carapace; therefore, the magnetic NPs do not affect their protein configuration, being only deposited onto the biomarker’s surface, as seen in Figure 3b. This is an important result, and it strongly suggests the application of this nanocomposite, for example, in As removal processes of real effluents, as previously tested with success [30].

The after-exposure ternary nanocomposite was recovered with the assistance of magnetic decantation, and the liberation of the NPs into the water after two weeks of interactions was completed with the *D. magna*.

**Figure 4:**
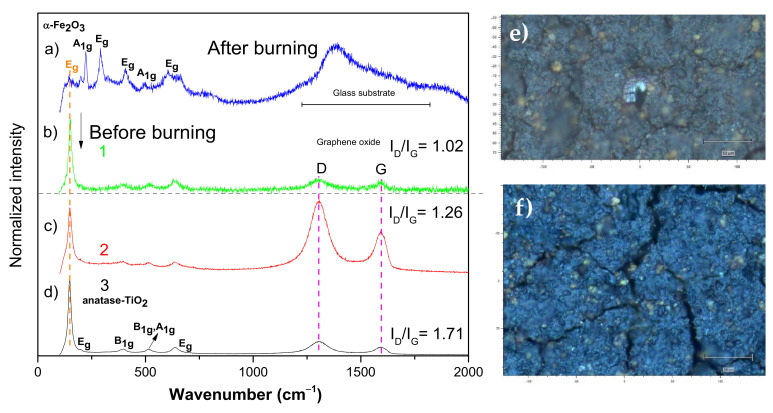
Before- and after-burning µ-Raman spectra for different spots in the ternary nanocomposite (**a**–**d**). (**e**) Note how the darker material becomes whitish after the burning due to the phase transition of the iron-oxide. (**e**) After-burning micrograph for the recovered ternary nanocomposite measured over a glass substrate. (**b**–**d**) 1, 2, 3 (before burning) correspond to Raman spectra taken by exciting bright, darker, middle (crack) spots, respectively in the picture (**f**).

We have also taken the wet recovered sample and put it into a glass substrate for measurement (paste form). The µ-Raman spectra, taken for the recovered wet magnetic r-GO composite (Figure 4a–d), only present the main Raman modes for the anatase. In addition, after burning the sample (see Figure 4a), the characteristic Raman modes of hematite have appeared, and the anatase Raman modes are also seen, in agreement with the micrograph in Figure 4e (white powder region).

However, the presence of the glass substrate made it difficult to characterize and confirm the r-GO presence. To resolve this issue, we characterize the recovered dried sample. As given in Figure 4f, three sections are observed including blue and white granular sections and black fissures, in which Raman spectra were recorded to find out possible differences in their structure and compositions. Two clear Raman modes for the D- and G-bands of the r-GO phase are positioned at 1306 and 1599 cm^−1^, respectively. As reported by Takur et al. [31], the D-band shows higher intensity than the G-band, confirming more isolated graphene domains in the case of r-GO. It happens for the three measured sections, and an increment in the intensity ratio (I_D_/I_G_) was also noticed. Values from 1.02 to 1.71 were calculated, and they are likely different due to the size distribution of nano γ-Fe_2_O_3_ and anatase growth on the GO surface. Furthermore, regions with more NP concentrations have shown high I_D_/I_G_ values, confirming that the inorganic synthesis procedure significantly influences the GO reduction, and more important, the r-GO configuration is kept even after the ecotoxicological test and recuperation. The nanocrystalline size (L_a_) of the r-GO of the sample can also be obtained by using the next relation [32]:

(1) $$L_{a}\left(\mathrm{nm}\right)=\frac{560}{E_{l}^{4}}{\left(\frac{I_{D}}{I_{G}}\right)}^{-1}$$

where E_l_ is the laser excitation energy (eV), with λ = 785 nm (E_l_ = 1.58 eV) [31]. We have obtained values of 88.1, 71.3, and 52.5 nm, respectively, for the three analyzed regions nominated above (blue and white granular sections and black fissures). The I_D_/I_G_ value for the pure GO phase was previously calculated, and it was found to be equal to 0.8, hence a value of L_a_ = 112 nm was calculated [29]. By comparing this with the L_a_ values (88.1, 71.3, and 52.5 nm) for the three regions, we can see a decrease in the size values for the ternary nanohybrid when compared with the pure GO phase. Thus, this result may be explained assuming the decrease in size of the sp^2^ domains with partial ordered cystallites [33].

### 3.2. Rietveld Refinement Analysis

**Figure 5:**
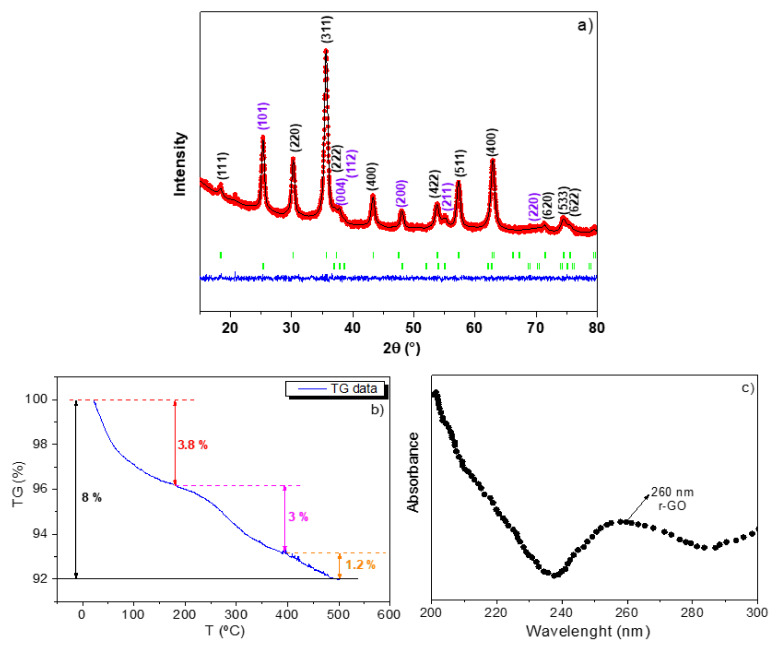
(**a**) Rietveld refinement of the XRD diffractogram of the ternary nanocomposite using the TCH diffraction profile. The red points (I_obs_) and the black lines (I_cal_) respectively represent the observed experimental diffractograms and the calculated diffractograms, and the blue line is the residual lines. Miller indices with black and purple colors indicate the crystallographic γ-Fe_2_O_3_ and anatase TiO_2_ phases, respectively, (**b**) TG curve, and (**c**) UV vis spectrum of the ternary nanocomposite.

Figure 5a displays the XRD pattern of the ternary nanocomposite with the corresponding refinement model, as described below. First, it can be seen that there are only two main crystalline phases, pure anatase TiO_2_ and γ-Fe_2_O_3_, i.e., the Bragg peaks of GO phase are not observed. In other words, there are two sets of peaks: a first set indexed to the (111), (220), (311), (222), (400), (422), (511), (400), (620), (533), and (622) Miller lattice planes of the γ-Fe_2_O_3_ phase and the (101), (004), (112), (200), (211), and (220) diffraction planes of the anatase TiO_2_ structure.

Using the modified Scherrer approach, the anisotropic size broadening can be written as a linear combination of spherical harmonics (SHP) available in the FullProf Suite software, and it is supposed that anisotropic size contribution only comes from the Lorentzian component of the total Voigt function [34]. Then, the explicit formula for the SPH treatment of size broadening is given by Equation (2) below and found in the literature [35,36]:

(2) $$\beta_{h}=\frac{\lambda }{D_{h}\cos\theta }=\frac{\lambda }{\cos\theta }\sum_{\mathrm{lmp}}a_{\mathrm{lmp}}y_{\mathrm{lmp}}\left(\Theta_{h}\Phi_{h}\right)$$

where h represents the (hkl) indices, $\beta_{h}$ is the size contribution to the integral breadth of reflection (hkl), $y_{\mathrm{lmp}}\left(\Theta_{h}\Phi_{h}\right)$ are the real components of spherical harmonics (arguments $\Theta_{h}$ and $\Phi_{h}$ are the polar and azimuthal angles of vector (hkl) with respect to a Cartesian crystallographic frame), and a_lmp_ are the refined coefficients, related to the Laue class (given in Table 1) [35]. For the γ-Fe_2_O_3_, an m3 Laue Class was used, while for the anatase TiO_2_, a 4/m Laue class was considered.

The refined diffractogram of the ternary nanocomposite is shown in Figure 5a. After the Rietveld refinement of the coefficients a_lmp_, the program estimates the average apparent crystallite size in Å. In addition, we have used the external IRF file of the standard corundum to subtract the instrumental broadening entering the parameters where the FullProf Suite software generated a mic file, which stores the anisotropic size values. The refined parameters, the percentage of phases in the nanocomposite, and global average crystallite sizes are summarized in Table 2. As it can be noticed, these data corroborate the presence of the inverse spinel γ-Fe_2_O_3_ and the corresponding tetragonal structure of the anatase phase in the ternary nanocomposite (no crystalline rutile phase contribution was detected). The mean apparent crystallite sizes obtained were 12.39 (6) nm and 11.71 (2) nm for the γ-Fe_2_O_3_ and anatase nanocrystallite phases, respectively. In addition, the quantitative Rietveld analysis for the ternary nanocomposite sample of the crystalline phases gave percentage values of 72% and 28% for the γ-Fe_2_O_3_ and anatase, respectively. These results agree to the previous microstructural values before ecotoxicological experiments.

### 3.3. TG and UV-Vis Analysis

The TG curve for the ternary nanocomposite depicts a total weight loss of 8%, as shown in Figure 5b. However, we can first see three clear stages: (i) one occurring between RT to 180 °C, (ii) the second one from 180 to 390 °C, and (iii) the third one from 390 to 500 °C. Experimentally, no extra weight loss was detected. According to Nurdin et al. [37], the first and second stages are intrinsically related to the evaporation of the adsorbed water and crystalline water. Nevertheless, it should be mentioned that the second stage is assigned to the water-critical temperature of 374 °C (bonding water at the interface). For the γ-Fe_2_O_3_ NPs stabilized in the presence of diverse concentrations of nitric acid, the stabilization temperature varies in the interval from 425 to 550 °C when decreasing the nitric acid molarity [37]. In our case, the γ-Fe_2_O_3_ NPs are not effectively coated by the two other phases, but the presence of TiO_2_ anatase and r-GO has favored the stabilization of the γ-Fe_2_O_3_ phase in high temperatures. In fact, a similar trend for the weight loss in the rGO–anatase-TiO_2_ hybrid was reported in the literature [37], where the stabilization temperature is reached at ~500 °C, which, as explained, was also the case for the last third of weight loss observed in our sample.

The UV-Vis spectrum for the ternary nanocomposite showed two defined regions, as seen in Figure 5c. The first one is related (200–240 nm) to the descendent curve (broad absorbance lines) of the metal-iron oxide/graphene nanocomposite [38,39]. The reduction in graphene has often been studied by several functional groups, including sugar derivates and aqueous phytoextracts [30,32]. Based on µ-Raman measurements, the surface modification of GO during metal oxide growth yielded to the reduction in GO sites due to the occupancy of the oxygen coordinating with the trivalent irons. More important, based on the obtained UV-Vis spectrum, we can clearly observe a broad peak located at 260 nm. This red shift (232 nm, the π–π* transitions of the aromatic C–C bonds) confirms the inorganic reduction from the original GO, as also reported in the literature in [31,33].

### 3.4. Morphological, Structural, and Chemical Characterization by TEM, EDS, and EELS

**Figure 6:**
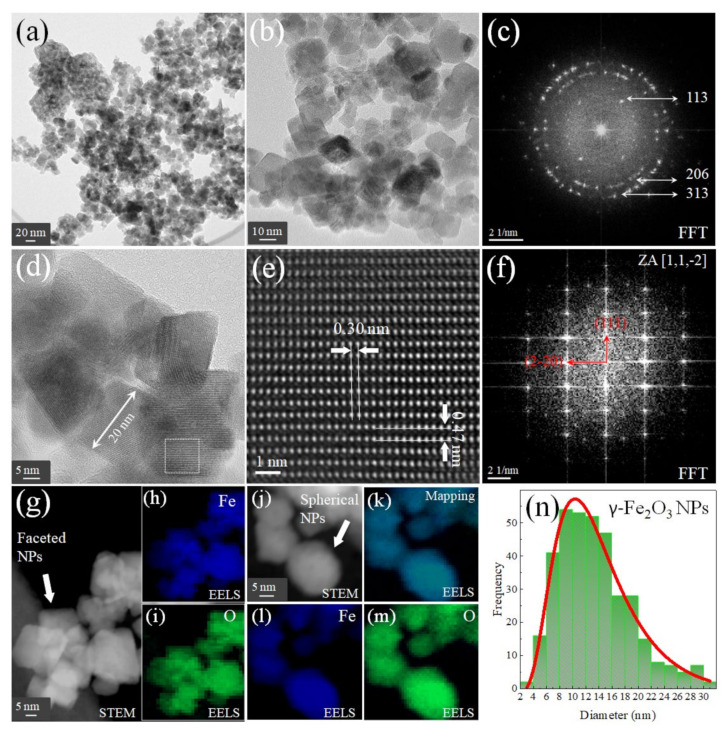
(**a**,**b**) TEM images of the γ-Fe_2_O_3_ NPs, (**c**) the FFT of (**b**,**d**) high-resolution image, (**e**) the magnification of the white box in (**d**,**f**), FFT of (**e**,**g**) is the STEM image for faceted NPs and (**h**), and (**i**) the EELS images for Fe and O taken from (**g**). On the other hand, (**j**) is the STEM image for spherical NPs with their respective EELS mapping (**k**) and elemental identification for Fe (**l**) and O (**m**). (**n**) is the PSD for γ-Fe_2_O_3_ NPs considering both morphologies.

**Figure 7:**
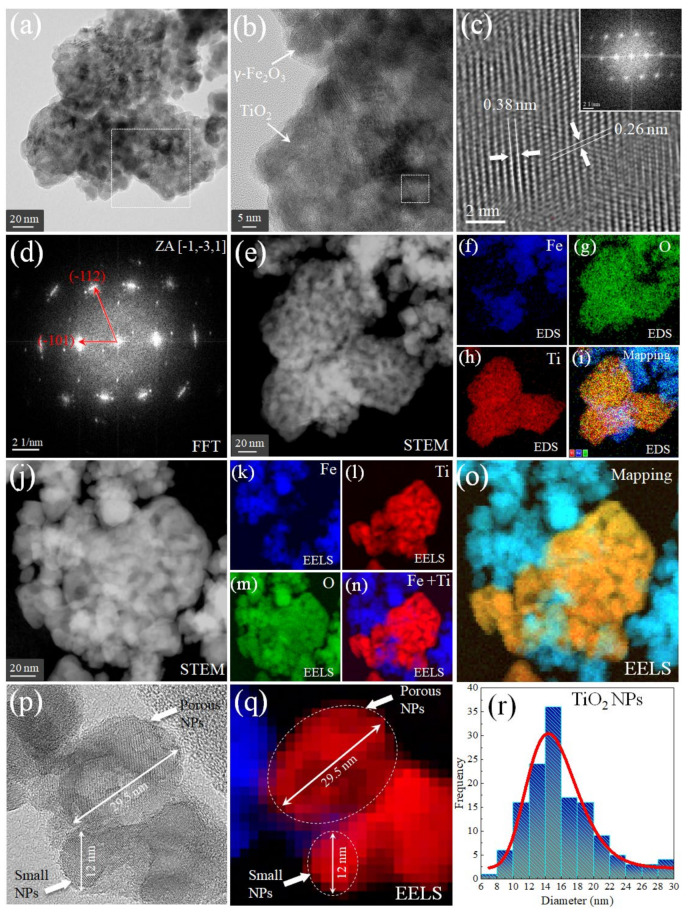
(**a**) TEM image for the nanocomposite, (**b**) magnification of the white box in (**a**) where the γ-Fe_2_O_3_ and TiO_2_ NPs are observed (white arrows), (**c**) high-resolution magnification of (**b**) where the FFT was taken, see right top inset of (**c**), (**d**) FFT of Image (**a**), while (**e**) is the STEM image of (**a**,**f**–**i**), which are elemental EDS images for Fe, O, Ti, and mapping. (**j**) STEM image of another selected region from the sample with (**k**–**n**) representing EELS images for the Fe, Ti, O, and Fe+ Ti, where (**o**) is the total EELS mapping. (**p**) is the high-resolution image of the ternary nanocomposite, (**q**) the EELS image, and (**r**) is the PSD for the TiO_2_ NPs.

Figure 6 and Figure 7 show statistical analysis of several TEM images of the nanocomposite. The NPs’ size distribution (PSDs) was calculated from histograms after counting approximately 500 NPs. The PSDs are highly asymmetric and are well represented by log-normal functions [40]. The obtained parameters are summarized in Table 3, where 〈*D*〉 is the mean NPs size, *D_m_* is the mode, ∆ is the standard deviation, and *D*_s_ is the dimensionless skewness.

From Figure 6a,b, we were able to distinguish the isolated γ-Fe_2_O_3_ phase at a different scale. The fast Fourier transform (FFT) was taken in Figure 6b, and it is indexed in Figure 6c, where the nano polycrystalline' nature is exhibited by the characteristic rings (different orientations and interplanar distances). Moreover, it is important to mention that faceted-like morphologies were observed (see Figure 6d). This is an indirect indication of the presence of GO in the sample, which in this case is acting like a size/shape controlling agent, as reported in previous works [29,30,41]. In addition, considering the small GO concentration (2 mg mL^−1^) in the ternary nanocomposite synthesis [30], it can be inferred that this GO quantity was not sufficient to result in a full regular geometry. On the other hand, the FFT extracted from Figure 6d exhibited a singlecrystal configuration (Figure 6e,f), where the characteristic γ-Fe_2_O_3_ interplanar distances of 4.8 Å and 3 Å were observed for the (111) and (220) Miller planes assigned to random vacancies space group Fd$\bar{3}$m [42]. Figure 6e also shows a HRTEM image of the traced area in Figure 6d belonging to an individual NP. The corresponding FFT from the image of Figure 6d is shown in Figure 6f. The spot pattern in the FFT was automatically indexed using JEMS software (P. Stadelmann CIMEEPFL, Jongny, Switzerland) using the cubic (Fd$\bar{3}$m) phase of the γ-Fe_2_O_3_ crystal structure. Most spots from the pattern that represents a crystalline domain can be indexed in the [1,1,−2] direction of the γ-Fe_2_O_3_. The STEM image of faceted NPs (see Figure 6g) shows a mesoporous character, a feature that allows for explanations of the high arsenic removal efficiency previously studied [30]. The EELS images for Fe and O (see Figure 6h,i) confirm the homogeneous distribution of both elements in the inverse spinel. The same elemental configuration is observed in Figure 6j–m for the spherical γ-Fe_2_O_3_ NPs, suggesting that the samples kept their morphologies even after the As removal process and after performing ecotoxicological experiments. Finally, it was possible to build the PSD graph by counting all morphologies depicting a mean particle size of 14.3 nm, as seen in Table 3.

By looking into Figure 7a,b, it was possible to demonstrate that in the ternary nanocomposite the γ-Fe_2_O_3_ and TiO_2_ NPs are independently distinguished, and Figure 7d depicts the nanocrystalline nature of the ternary nanocomposite. In Figure 7c, a magnification and FFT were performed to clearly study the interplanar distance and monocrystal phase of the anatase TiO_2_, where the 3.8 Å and 2.6 Å distances were estimated. These values are characteristic of the I4_1_/amd space group of the anatase TiO_2_ phase [43]. The STEM image (obtained in the region where Figure 7a was recorded) confirms the mesoporous-like configuration for the ternary nanocomposite, as shown in Figure 7e. The elemental EDS images in Figure 7f–h and mapping partially allowed for differentiations between both metal oxide phases. Hence, the STEM image was collected in another region of the sample, Figure 7j, and the elemental EELS analysis was again performed (Figure 7k–n). Thus, the final EELS mapping (sum of independent elemental contributions in Figure 7o) clearly distinguished both phases; therefore, it is possible to estimate the mean size for the anatase TiO_2_ NPs.

From the above results, it can be inferred that it is important to perform EELS to be capable of differentiating different metal oxides in this kind of ternary nanocomposite. In addition, this experiment will allow for double statistical counting and reduce bad size estimation of the right NPs when they have similar morphologies, a known critical issue in diverse applications. Moreover, the high-resolution images in Figure 7p and the elemental EELS in Figure 7q confirmed the presence of porous and small TiO_2_ NPs, whose PSD distribution is given in Figure 7r, showing a mean particle size of 15.5 nm, in close agreement with the Rietveld analysis.

**Figure 8:**
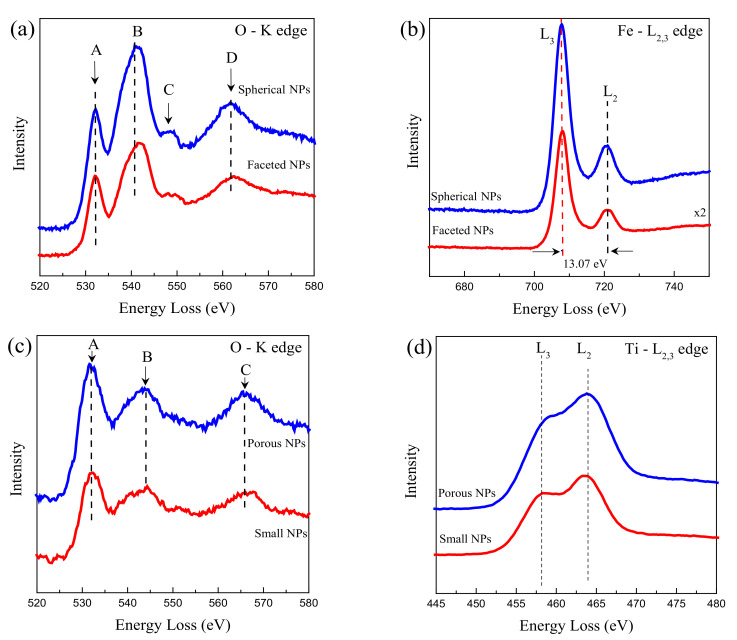
(**a**) O-K edge and (**b**) Fe-L_2,3_ edge in the EELS spectra of the spherical and faceted γ-Fe_2_O_3_ NPs identified in Figure 6g,j. (**c**) O-K edge and (**d**) Ti-L_2,3_ edge in the EELS spectra of the porous and small TiO_2_ NPs identified in Figure 7p,q.

The elemental EELS technique is often employed to differentiate diverse metal oxides and valence states presented in the sample [44]. Conversely, the L_3_/L_2_ ratio is intrinsically related to the oxidation states [44]. Figure 8a,b depicts the O-K edge and Fe L_2,3_ edge for spherical and faceted γ-Fe_2_O_3_ NPs, while Figure 8c,d shows the O-K edge and Ti L_2,3_ edges for the porous and small TiO_2_ NPs. The maxima peaks for Fe are located at 708 and 721 eV with a separation of 13.07 eV, as reported in the literature for trivalent iron-oxides [45,46]. The location of both peaks was observed to be independent of the morphology. The L_3_/L_2_ ratio was found to be equal to 4.1 for the spherical and 4.3 for the faceted morphologies, respectively. These values are in agreement with previous Fe^3+^ states in nano iron-oxides previously reported [44,46]. The O-K edge showed four featured peaks for the spherical NPs: (1) a peak at 532 eV, (2) the intense peak at 540 eV, (3) a small shoulder at 549 eV, and (4) a broad contribution at 562 eV. The peaks in 1, 2, and 4 are characteristic of a high oxidation state [44] in the γ-Fe_2_O_3_ NPs, while the shoulder at 549 eV can originate from transitions in the O-2p states to Ti 4s/4p orbitals (often observed in the interval from 540 to 549 eV) [47]. This means that some traces from anatase TiO_2_ NPs remained even in this isolated γ-Fe_2_O_3_ phase, but in less proportion in the faceted NPs. Additionally, no contributions from the oxygen groups of graphene oxide were observed, confirming its reduction.

For completing the chemical analysis of the ternary nanocomposite, we studied the O-K edge and Ti L_2,3_ edge EELS spectra for the anatase TiO_2_ NPs, which are shown in Figure 8c,d. Three featured peaks in the O-K edge are detected at (A) 532 eV, (B) 544 eV, and (C) 566 eV for both porous and small TiO_2_ NPs. All the peaks correspond to the anatase TiO_2_ NPs [48]. Rutile is often characterized by three additional peaks located after the A and B peaks, which was not observed in the EELS spectra [48]. When examining the Ti edge EELS spectra for porous and small TiO_2_ NPs, two marked L_3_ and L_2_ transitions are observed, and they are quite similar in shape with the energy loss position at 458 and 464 eV, characteristic values also found for anatase TiO_2_ NPs [48].

### 3.5. Mössbauer and VSM Measurements

**Figure 9:**
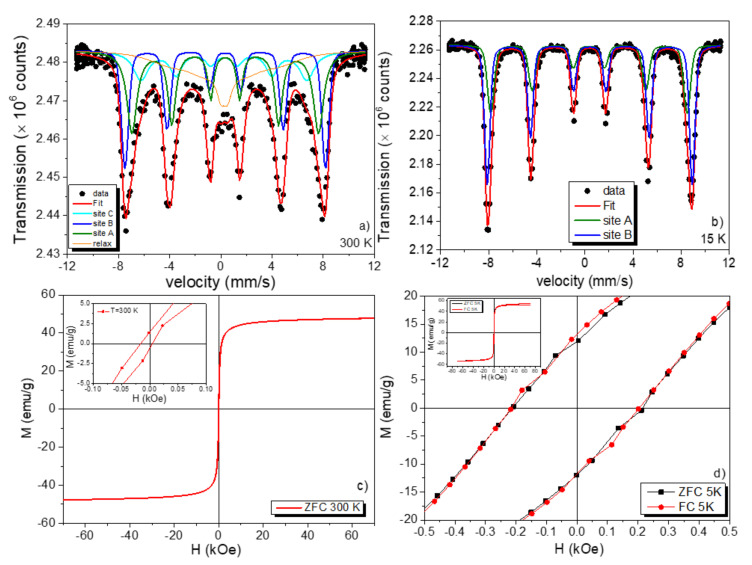
(**a**) 300 K and (**b**) 15 K Mossbauer spectra for the ternary nanocomposite, (**c**) ZFC 300 K *M*(*H*) loop and (**d**) ZFC and FC 1T *M*(*H*) loop recorded at 5 K for the ternary nanocomposite. 1 T was the field used in the FC protocol when the sample was cooled down from 300 K to 5 K. Insets in (**c**) and (**d**) are zoomed regions of their respective *M*(*H*) loops.

Considering that the ternary nanocomposite has the γ-Fe_2_O_3_ phase and the ^57^Fe Mössbauer spectroscopy measures the hyperfine interactions that allow us to distinguish the different Fe configurations and phases and also their thermal and chemical stability, we have performed zero-field ^57^Fe Mössbauer measurements at RT and 15 K for the recuperated ternary nanocomposite after ecotoxicological tests, and the results are shown in Figure 9a,b. The RT ^57^Fe Mössbauer spectrum was fitted using four magnetic components. The two static magnetic sextets (A and B) are often found in the γ-Fe_2_O_3_ phase. These two sextets (A and B) have mean values for the isomer shift 〈δ〉 equal to 0.24 and 0.34 mm/s, and mean magnetic hyperfine field (B_hf_) values of 47.8 T (site A, olive color) and 48.7 T (site B, blue color), respectively. The other two components [third (3rd) and fourth (4th) subspectra] were respectively used to account for the Fe^3+^ spins located at the NP surfaces (3rd-component) and Fe^3+^ in very small particles that are entering in the superparamagnetic regime at RT (4th-component), i.e., with significant overbarrier fluctuations (jumping time rates of 10^−12^ s). For the 4th-component, a Blume–Tjon-level model was applied, revealing values of B_hf_ = 33 T and 〈δ〉 = 0.27 mm/s, with a relative absorption area (RAA) of 23%. This RAA value is in close agreement with the one obtained before exposure (RAA~20%) to *D. magna* [29]. In this regard, it confirms that, structurally speaking, no modification was found in the 19.6 nm ternary nanocomposite. Moreover, this RT spectrum is similar in shape with that obtained before the exposition process; consequently, its main contribution can be assigned to nanoparticles with well-defined ordered crystallites and sizes bigger than 10 nm. The fitted 15 K Mössbauer spectrum only exhibits the two static asymmetric sextets of the γ-Fe_2_O_3_ found for the spinel sites in the Fd$\bar{3}$m cubic structure. Thus, it indicates that the spin relaxation effect, observed strongly in the 4th-component, has been reduced due to the temperature reduction, and consequently, both the very small γ-Fe_2_O_3_ NPs (4th-component) and the Fe^+3^ spins from the NP surfaces (3rd) are now at 15 K in a magnetically blocked state (they overlap with the conventional A and B sites of the spinel structure and there is no energy resolution to distinguish this fraction by zero-field Mössbauer experiments- usually these phases can only be split by in-field ^57^Fe Mössbauer experiments). Thus, the above results help us to infer about the quality of the γ-Fe_2_O_3_ NPs, before and after the exposition time, with the effluent containing *D. magna*.

In addition, it should be mentioned that not only the hyperfine parameter values of ${\langle \delta \rangle }_{A}$ = 0.34 mm/s and ${\langle \delta \rangle }_{B}$ = 0.41 mm/s, with B_hf,A_ = 50.7 T, and B_hf,B_ = 52.9 T, are close to those values of the bulk γ-Fe_2_O_3_ phase, the RRA values are also equal to those found in the bulk-like phase (36.9% for the site A and 63.1% for the site B [9,29]); consequently, these findings are finger prints for well-crystallized and chemically stable γ-Fe_2_O_3_ NPs in the ternary nanocomposite even after being exposed and recovered from the ecotoxicological assays. These results also agree with the EELS analysis discussed above. To understand a bit more about the magnetic properties of the nanocomposite (^57^Fe Mössbauer only gave us the Fe magnetically blocked state, and the magnetic state can only be obtained with in-field experiments) after the exposure experiments, we have measured *M*(*H*) loops at RT and 5 K, and the results are displayed in Figure 9c,d. First, the magnetization data revealed values for saturation magnetization of 47.7 emu g^−1^ at RT and 55 emu g^−1^ at 5 K, which are again in close agreement with the magnetization values before exposure to *D. magna* [15]. ZFC and FC 1T *M*(*H*) loops reported the same *M_s_* value, where non-minor hysteresis loops were observed under the present protocol. In Figure 9c, the coercivity field (*H_C_*) shows the expected temperature behavior, but with a value ca. 9.5 Oe at RT, indicating that the ferrimagnetic γ-Fe_2_O_3_ NPs can be considered as a soft magnetic material. Moreover, the 5K ZFC/FC *M*(*H*) loops have the same *H_c_* fields, with a value of 204 Oe, indicating that there is no exchange bias effect as well.

Finally, before spreading this nanomaterial into the real body’s waters, the *LC*_50_ values must be determined, as this will reveal insights into the suitable concentrations for toxic metal removal experiments. Moreover, protocols for the recuperation and recyclability of these nanomaterials are lacking in the literature, and it was briefly discussed in this manuscript. Few reports of biomarkers’ ecotoxicity are presented for metal and metal oxide NPs [15,49,50]. However, for binary and ternary nanocomposites (which have been tested to increase toxic metal uptake), there is not enough information to have a total ecotoxicological profile of these magnetic nanocomposites. In addition, post-exposure characterization must be performed on the recovered nanohybrids after heavy metal removal or ecotoxicological experiments. Thus, this will guide researchers about novel protocols of recuperation, storage, and further reuse.

## 4. Conclusions

A magnetic r-GO supporting anatase and γ-Fe_2_O_3_ NPs nanocomposites has been synthesized by the co-precipitation method and used for ecotoxicological experiments on *D. magna* at two exposure doses (*LC*_50_ of 0.5 and 1 mg mL^−1^). The ternary nanocomposite, deposited onto a *D. magna* carapace, was studied by µ-Raman spectroscopy, which elucidated that no changes have occurred in the structural properties of the ternary nanocomposite. However, it is important to highlight that checking the interaction of the nanocomposite with the protein environment of the biomarker, it was not possible to detect the Raman vibration modes for the *D. magna* even after the individuals have been isolated over a glass substrate. To corroborate any possible damage at the molecular level, an electrophoresis DNA separation protocol was used, revealing similar concentrations of DNA proteins for the negative control and for the *LC*_50_ dose. This result confirmed that the ternary nanocomposite likely affects the external body of the biomarker, but subsequent studies on this issue are still required considering that gene sequences for *D. magna* still need to be identified. The employed protocol allowed us to characterize the structural and vibrational properties of the nanocomposite, where the three phases of the nanomaterial did not show any modification in their chemical composition and physical properties when exposed to the *D. magna* and the effluent as well. More specifically, using an easy magnetic decantation process the magnetic ternary nanocomposite was recuperated and further characterized by distinct analytical methods. Notoriously, the Rietveld analysis gave close values for the mean crystallite domains of the γ-Fe_2_O_3_ and anatase crystallographic phases before and after the ecotoxicological properties. More important, the r-GO is favored by growing inorganic NPs onto GO. TEM, EDS, and EELS techniques permitted correctly distinguishing both metal oxide phases and hence the accurate determination of their mean sizes and morphological properties. So, two important results can be highlighted (i) the nanocomposite can be reused, and (ii) it does not significantly affect the *D. magna* individuals. The thermal stability of the ternary nanocomposite, where the γ-Fe_2_O_3_ phase is predominant, was also studied, resulting in a stable temperature of 500 °C for the total nanocomposite. This r-GO phase is also kept even after exposure to the ecotoxicological experiments and further recovery for storage, as demonstrated by the I_D_/I_G_ = 1.71 value and the redshift to 260 nm in the UV-Vis spectrum, and as a supporting framework to conserve the valence state of iron-spins and their magnetic properties of the γ-Fe_2_O_3_ NPs as studied by EELS, Mössbauer, and magnetization experiments at RT and low temperatures (15 and 5 K). Finally, all these results suggested that the magnetically recovered ternary nanocomposite can be still reused for other future applications, and the preliminary results indicate that the nanocomposite, already tested for the As removal process, does not affect significantly the *D. magna* species.

## Figures and Tables

**Table 1:** DNA concentrations obtained from the gel electrophoresis test after exposure to the ternary nanocomposite (*LC*_50_ value) and its comparison to the negative control.

	Ternary Nanocomposite (0.55 mg mL^−1^)			Negative Control		
	A1	B1	C1	A2	B2	C2
**Concentration ng mL^−1^**	60	30	15	60	30	15

**Table 2:** Rietveld refinement parameters of the ternary nanocomposite using FullProf Suite program: cell lattice parameters, cell volume, Caglioti parameters, and agreement factors. Rp (%) (profile residual) and Rwp (%) (weighted profile residual). Goodness of fit, chi-square (χ^2^).

Refinement Parameters	Ternary Nanocomposite	
	γ-Fe_2_O_3_	Anatase
$a\;\left(Å\right)$	8.356	3.787
$b\;\left(Å\right)$	8.356	3.787
$c\;\left(Å\right)$	8.356	9.515
$\alpha \;\left(Å\right)$	90	90
$\beta \;\left(Å\right)$	90	90
$\gamma \;\left(Å\right)$	90	90
$V\;\left({Å}^{3}\right)$	583.4 (3)	136.5 (2)
$K_{00}$ $,\;Y_{00}$	−0.306 (6)	−5.692 (4)
$K_{41}$ $,\;Y_{20}$	−0.260 (2)	2.507 (8)
$K_{61}$ $,\;Y_{40}$	0.623 (1)	−1.834 (8)
$K_{62}$ $,\;Y_{44+}$	1.191 (1)	−1.533 (6)
$K_{81}$ $,\;Y_{44-}$	0.532 (7)	0.634 (2)
$Y_{60}$		0.087 (5)
$Y_{64+}$		0.461 (4)
$Y_{64-}$		2.142 (3)
FWHM parameters		
U	0.0041	4.3586
V	−0.0829	−2.6837
W	0.0311	0.9158
Average max strain	16.3 (6)	154.2 (0)
Average size (nm)	12.4 (6)	11.7 (2)
phase percentage (%)	72.3	27.7
$R_{p}\;\left(\%\right)$	13.7	
$R_{wp}\;\left(\%\right)$	8.29	
χ^2^	1.10	

**Table 3:** Values extracted from the lognormal fitting to the histograms.

NPs	〈D〉 (nm)	$D_{m}$ (nm)	∆ (nm)	Ds
γ-Fe_2_O_3_	14.31	11.53	7.02	1.59
TiO_2_	15.42	14.68	3.45	0.68

## Data Availability

The original data related to this research can be asked for any time at to the corresponding author’s email: juan.ramos5@unmsm.edu.pe.
